# Correction: Matsutake et al. Fast and Stable Responses during Decision Making Require Strong Inhibitory Processes in Soccer Players. *Brain Sci.* 2024, *14*, 199

**DOI:** 10.3390/brainsci15020193

**Published:** 2025-02-14

**Authors:** Takahiro Matsutake, Hiroki Nakata, Genta Matsuo, Takayuki Natsuhara, Kisho Zippo, Kouki Watanabe, Takayuki Sugo

**Affiliations:** 1 Research Center for Urban Health and Sports, Osaka Metropolitan University, Osaka 558-8585, Japan; 2 Faculty of Engineering, Nara Women’s University, Nara 630-8263, Japan; 3 Department of Sport Sciences, Osaka University of Health and Sport Sciences, Osaka 590-0459, Japan; 4 Faculty of Applied Psychology, Tokyo Seitoku University, Chiba 276-0013, Japan; 5 Department of Sport Medicine and Research, Japan Institute of Sports Sciences, Japan High Performance Sport Center, Tokyo 115-0056, Japan; 6 Graduate School of Sport and Exercise Sciences, Osaka University of Health and Sport Sciences, Osaka 590-0459, Japan

In the original publication [1], an error occurred in the presentation of Table 4. Specifically, Table 4 intended to describe the peak latencies and amplitudes of the subtracted No-go N2 and P3. However, the results as presented were incorrect because they failed to reflect that P3 is a positive potential, as accurately outlined in the Statistical Analysis section. Below, the corrected Table 4 is provided. The authors state that the scientific conclusions are unaffected. This correction was approved by the Academic Editor. The original publication has also been updated.

## Figures and Tables

**Table 4:** Peak latencies and amplitudes of subtracted No-go N2 and P3.

		Go/No-Go Task			Pass Choice Reaction Task		
Electrode		Novice	Low	High	Novice	Low	High
**Subtracted No-go N2 Amplitude (μV)**	**Fz**	−6.5 ± 5.3	−11.8 ± 5.3	−12.3 ± 6.9	−3.8 ± 3.8	−9.3 ± 6.1	−11.4 ± 3.7
	**F3**	−9.2 ± 4.6	−14.0 ± 4.2	−13.9 ± 5.3	−3.9 ± 3.1	−9.3 ± 5.8	−11.1 ± 5.0
	**F4**	−9.4 ± 4.9	−13.3 ± 1.9	−13.3 ± 4.7	−5.1 ± 3.8	−8.2 ± 3.5	−9.5 ± 2.5
**Subtracted No-go N2 Latency (ms)**	**Fz**	287 ± 17	285 ± 23	282 ± 53	343 ± 31	336 ± 24	320 ± 19
	**F3**	286 ± 17	287 ± 25	285 ± 50	339 ± 27	341 ± 25	322 ± 20
	**F4**	281 ± 38	284 ± 20	290 ± 66	347 ± 36	336 ± 25	319 ± 38
**Subtracted No-go P3 Amplitude (μV)**	**Fz**	20.4 ± 9.2	15.5 ± 9.3	17.2 ± 10.1	11.0 ± 8.0	11.2 ± 8.9	12.3 ± 6.1
	**F3**	16.4 ± 7.0	9.7 ± 10.4	13.4 ± 8.7	10.6 ± 8.0	7.8 ± 7.2	10.1 ± 4.5
	**F4**	15.3 ± 7.3	12.7 ± 8.0	14.5 ± 9.5	6.6 ± 5.3	9.8 ± 6.8	11.4 ± 5.5
**Subtracted No-go P3 Latency (ms)**	**Fz**	425 ± 90	388 ± 19	397 ± 18	448 ± 33	464 ± 47	445 ± 24
	**F3**	421 ± 90	392 ± 23	388 ± 16	506 ± 69	453 ± 34	430 ± 30
	**F4**	430 ± 85	407 ± 42	404 ± 16	415 ± 119	462 ± 39	425 ± 104

The data are expressed as mean ± SD.

## References

[1] Matsutake T., Nakata H., Matsuo G., Natsuhara T., Zippo K., Watanabe K., Sugo T. (2024). Fast and Stable Responses during Decision Making Require Strong Inhibitory Processes in Soccer Players. Brain Sci..

